# Clinical Application of the PES/WES Index on Natural Teeth: Case Report and Literature Review

**DOI:** 10.1155/2017/9659062

**Published:** 2017-02-05

**Authors:** Alessandro Lanza, Fabrizio Di Francesco, Gennaro De Marco, Felice Femiano, Angelo Itro

**Affiliations:** ^1^Multidisciplinary Department of Medical, Surgical and Dental Sciences, Campania University Luigi Vanvitelli, Via Luigi De Crecchio 7, 80138 Naples, Italy; ^2^Dental Prosthesis and Implantology, Multidisciplinary Department of Medical, Surgical and Dental Sciences, Campania University Luigi Vanvitelli, Via Luigi De Crecchio 7, 80138 Naples, Italy

## Abstract

The use of reliable indices to evaluate the aesthetic outcomes in the aesthetic area is an important and objective clinical aid to monitor the results over time. According to the literature various indices were proposed to evaluate aesthetic outcomes of implant-prosthetic rehabilitation of the anterior area like Peri-Implant and Crown Index [PICI], Implant Crown Aesthetic Index [ICAI], Pink Esthetic Score/White Esthetic Score [PES/WES], and Pink Esthetic Score [PES] but none of them was related to prosthetic rehabilitation on natural teeth. The aim of this study is to verify the validity of PES/WES index for natural tooth-prosthetic rehabilitation of the anterior area. As secondary objective, we proposed to evaluate the long-term predictability of this clinical application, one of which is presented below, following the analysis of the most currently accepted literature.

## 1. Introduction

A goal of modern dentistry is the placement of aesthetically pleasing restorative material. Single-tooth replacement in the anterior area presents a challenge for the clinician. In this region, treatment considerations include shape and shade matching of the crown, interdental spacing, topography of the ridge, contacts of the opposing dentition, parafunctional habits, and esthetic desires of the patient. Facial aesthetics is based on the harmony of both smile and face [1]. Fundamental parameters for an aesthetic smile are the position of the lips, gingival tissue condition, color, shape, and teeth position. Then when a prosthetic rehabilitation has been planned, each of the above-mentioned parameters should be performed [2, 3]. Careful rehabilitation plan and knowledge of the characteristics of the natural dentition are necessary for the rehabilitation of the maxillary anterior teeth. Clinical and radiographic examinations, study of models through diagnostic waxing, and cooperation with other specialists may be the key to have a high aesthetic and functional success [4–7]. The success of a single restoration in the esthetic zone depends mainly on the harmonious integration of the restoration into the patient's overall appearance, especially the peri-implant soft tissue [8, 9].

Both subjective (patients' ratings) and objective (esthetic scores and indices) assessments of implant esthetics are subject to growing interests [10, 11].

In order to achieve satisfactory outcome, it is essential to choose proper materials and techniques, whose quality has improved in dentistry. As a matter of fact, nowadays, zirconia ceramic systems are available; they have both biomechanical and mimetic high-quality properties that allow the clinician to achieve aesthetic and long-lasting results [12]. Longitudinal clinical studies using this system in anterior and posterior teeth show supportive outcomes, proving it can be an alternative to metal-ceramic fixed prostheses [13–15].

This case report presented an anterior zirconia ceramic's fixed prosthesis with changes in size, proportion, shape, color, and texture that prejudiced the smile's harmony. The Visual Analogue Scale (VAS) was recommended as a subjective measure of the esthetic outcome of implant-supported restorations [16].

Other means to assess the esthetic outcome of single-implant-supported restorations are various indices, such as implant aesthetic crown index (ICA), subjective esthetic score (SES), Peri-Implant and Crown Index (PICI), and comprehensive index comprising Pink and White Esthetic Score (PES/WES) [17–20]. Fürhauser et al. introduced an excellent index termed Pink Esthetic Score (PES) for evaluation of the soft tissue around single-implant crowns that might change over time; PES could be a useful tool for monitoring long-term soft tissue alterations [18]. Belser et al. [19] have later introduced Pink Esthetic Score (PES) to evaluate the esthetic outcome of soft tissue around implant-supported single crowns in the anterior zone and White Esthetic Score (WES) to specifically focus on the visible part of the implant restoration itself. The effects of the observer's specialization were further investigated in the study by Cho et al. [21] using PES/WES index, which was the only study to recruit periodontists. However, the study had limitations due to the small number of the examiners from each specialty group and no oral surgeons involved in the study. Meijer et al. [17] proposed the Implant Crown Aesthetic Index (ICAI) for evaluation of single-implant supported crowns. The limitations of the study by Meijer et al. were the small sample size and recruitment of only two specialists (oral surgeon and prosthodontist). According to a recent study comparing the indices and their reproducibility, PES/WES and PICI seemed to be more suitable than ICAI as esthetic indices for single-implant-supported crowns [20]. The main aim of this study is to describe the PES/WES index and its clinical application on natural teeth. PES/WES index is one of the most reliable prosthetic criteria to be followed in case of aesthetic rehabilitations in the frontal maxillary sector not only on dental implants but it can be used for natural teeth. We also proposed to evaluate the long-term predictability of this clinical application, one of which is presented below, following the analysis of the most currently accepted literature.

## 2. Case Presentation

**Figure 1:**
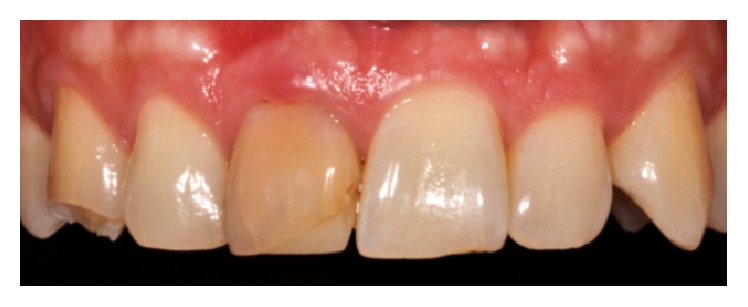
PES/WES started score: 5/10.

A 48-year-old female patient presents aesthetic problems related to the condition of the hard and soft tissue in the frontal maxillary sector (Figure 1), in particular coronal fracture of 1.3, abnormality of shape, volume and color of 1.1, asymmetry of the gingival margin with relative height, and volume alteration of mesial and distal papilla. Considering the clinical and radiographic preoperative exams, we analyzed clinical case using the index PES/WES parameters. The authors [19] have described the PES/WES index that combines both white and rose aesthetics parameters. In contrast to the original proposal [18], the PES comprises the following five variables (Table 1): mesial papilla, distal papilla, curvature of the facial mucosa, level of the facial mucosa, and root convexity/soft tissue color and texture at the facial aspect of the site. The WES specifically focuses on the visible part of the restoration itself and is based on the five following parameters: general tooth form; outline and volume of the clinical crown; color, which includes the assessment of the dimension's hue and value; surface texture; and translucency and characterization (Table 1). All ten parameters are assessed by direct comparison with the contralateral tooth and a score of 2, 1, or 0 is assigned to all ten parameters. Thus, a maximum total PES/WES of 20 can be reached which represents the optimum condition of the hard and soft tissues of the rehabilitated site compared to the characteristics of the contralateral natural tooth.

**Figure 2:**
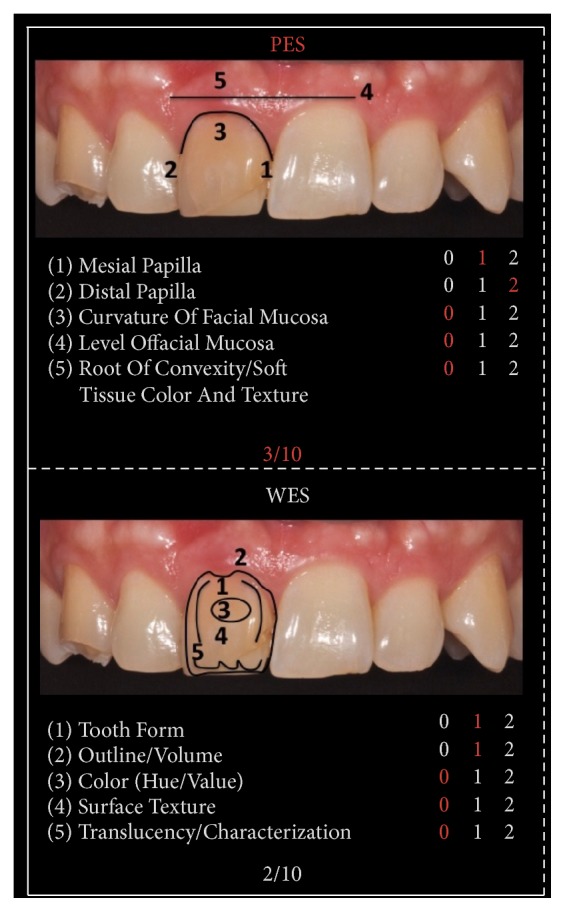
Initial PES/WES.

To determine PES and WES, crown on 1.1 was evaluated clinically and was photographed with the contralateral tooth. The initial score is 5 as described in Figure 1, given by the addition of the PES (3/10) and the WES (2/10) as shown in Figure 2: the result of the PES is given by the incomplete presence of the mesial papilla (1/10), the complete presence of the distal papilla (2/10), and the major discrepancy of other parameters (0/10); the result of the WES is given by the minor discrepancy of tooth form (1/10), outline/volume (1/10), and the major discrepancy of other parameters (0/10). According to the patient we created and analyzed a study model with relative diagnostic wax-up that highlights what will be the advantages and disadvantages of the future prosthesis. The program includes the direct restoration of 1.3 using a composite resin body A2, lithium disilicate prosthetic crown on natural tooth 1.1. The crown was cemented with dual cement Variolink Esthetic using adhesive technique recommended by the manufacturer. The photographs were made using Nikon D90 and a 105 mm lens (AF micro Nikkor 105 mm 1 : 2.8 D, Nikon) with a ring flash (EM-140 DG, SIGMA-Nikon).

**Figure 3:**
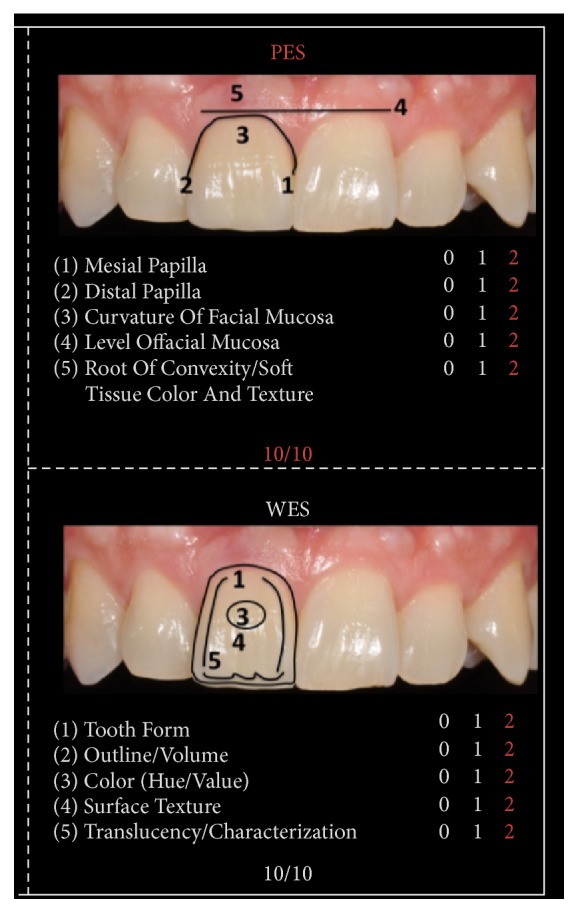
Final PES/WES.

**Figure 4:**
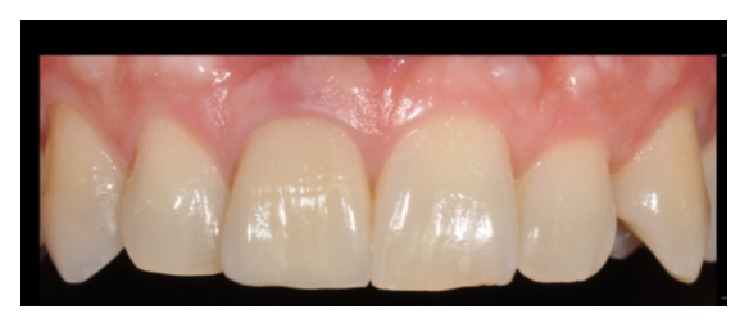
Crown just cemented. PES/WES final score: 20/20.

**Figure 5:**
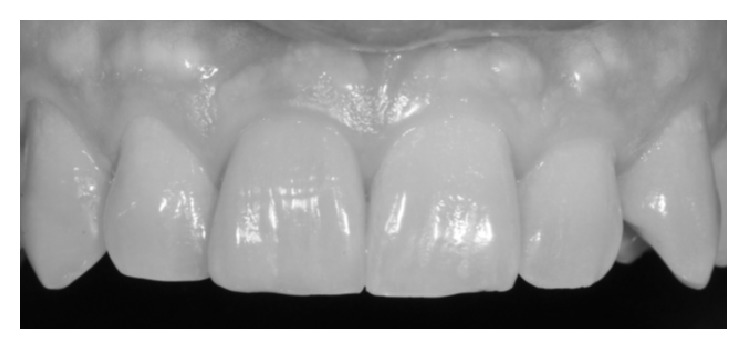
Black and white: checking value.

We found significant differences between the initial and final scores of the PES/WES or rather from 5 to 20 as described in Figures 3, 4, and 5. It is given by the addition of the PES (10/10) and the WES (10/10) as shown in Figure 3: the result of the PES is given by the complete presence of the mesial papilla (2/10), the complete presence of the distal papilla (2/10) and no discrepancy of curvature of facial mucosa (2/10), no discrepancy of level of facial mucosa (2/10), and no discrepancy of root convexity/soft tissue color and texture (2/10); the result of the WES is given by the no discrepancy of tooth form (2/10), no discrepancy of outline/volume (2/10), no discrepancy of color (hue/value) (2/10), no discrepancy of surface texture (2/10), and no discrepancy of translucency (2/10).

## 3. Discussion

**Figure 6:**
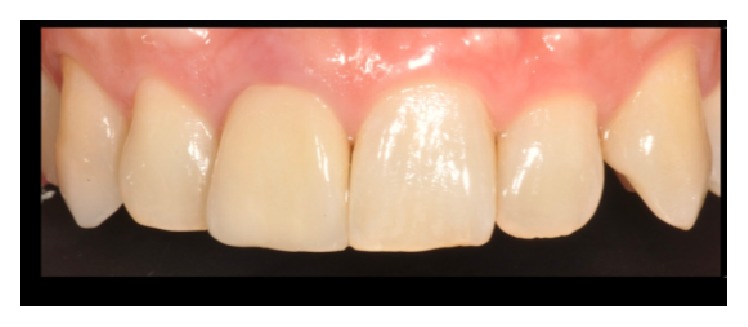
Follow-up of 5 years.

Nowadays, the aesthetic demands of patients are elevated, especially in visible areas such as the front region. According to the literature various indices were proposed to evaluate aesthetic outcomes of implant-prosthetic rehabilitation of the anterior sector. Belser et al. evaluated the esthetic outcome of maxillary anterior single-tooth implants using WES/PES, and they used the VAS to evaluate the satisfaction of the patient toward the single-implant in the esthetic zone [19]. We have reported in this case report a strong correlation between the esthetic evaluation performed by the dentist (PES/WES) and by the patient (VAS) as other studies have reported it [21, 22]. PES/WES like PICI seemed to be more suitable than ICAI as esthetic indices; they are reproducible esthetic indices that are not influenced by different observers and present similar outcomes in the overall esthetic evaluation and because of this, they are recommended for clinical use [20]. In this study we want to show how this index is reliable even for the aesthetic evaluation of hard and soft tissues in the prosthetic rehabilitation of the natural tooth and how this index can be a clinical aid in controlling the maintenance of pink and white tissue over time as shown in the follow-up of 5 years in Figure 6. As you can see the soft tissues were stable enough in time, whereas the white aesthetic parameters have had color changes and surface texture.

## 4. Conclusions

According to the literature about application of the PES/WES index to aesthetic evaluation of implant-prosthetic rehabilitation of the anterior sector, we also verified the validity of such index for natural tooth-prosthetic rehabilitation of the anterior area. The rightness of the PES/WES index for the objective outcome assessment of the esthetic dimension of anterior single-tooth crown was confirmed. However, prospective clinical trials are needed to further validate and refine this index and its clinical use also for natural tooth-prosthetic rehabilitation.

## Figures and Tables

**(a):** 

PES			
Parameter	Absent	Incomplete	Complete
(i) Mesial papilla	0	1	2
(ii) Distal papilla	0	1	2
	Major discrepancy	Minor discrepancy	No discrepancy
(iii) Curvature of facial mucosa	0	1	2
(iv)Level of facial mucosa	0	1	2
(v) Root convexity/soft tissue color and texture	0	1	2
Maximum total PES score			10

**(b):** 

WES			
Parameter	Major discrepancy	Minor discrepancy	No discrepancy
(i) Tooth form	0	1	2
(ii) Tooth volume/outline	0	1	2
(iii) Color (hue/value)	0	1	2
(iv) Surface texture	0	1	2
(v) Translucency	0	1	2
Maximum total WES score			10
